# Preparation of PBS/PLLA/HAP Composites by the Solution Casting Method: Mechanical Properties and Biocompatibility

**DOI:** 10.3390/nano10091778

**Published:** 2020-09-08

**Authors:** Muzamil Ahmad Khan, Zakir Hussain, Usman Liaqat, Muhammad Arman Liaqat, Muhammad Zahoor

**Affiliations:** 1 School of Chemical and Materials Engineering, National University of Sciences and Technology (NUST), Islamabad 44000, Pakistan; muzamil.phd@scme.nust.edu.pk (M.A.K.); armanliaqat786@gmail.com (M.A.L.); 2 Institute of Basic Medical Sciences, Department of Molecular Medicine, University of Oslo, Sognsvannsveien 9, 0768 Oslo, Norway; muhammad.zahoor@medisin.uio.no

**Keywords:** poly (l-lactic acid) (PLLA), poly (butylene succinate) (PBS), hydroxyapatite, nanocomposite, blend

## Abstract

The use of biodegradable polymeric scaffolds for tissue regeneration is becoming a common practice in the clinic. Therefore, an inclined trend is developing with regards to improving the mechanical properties of these scaffolds. Here, we aim to improve the mechanical properties of poly (butylene succinate) (PBS)/poly (l-lactic acid) (PLLA) blends by incorporating hydroxyapatite nanoparticles (HAP) in the blends to form composites. PBS/PLLA = 100/0, 95/5, 90/10, 85/15, and 0/100 wt% blends, along-with the loadings of a few mg of HAPs, were prepared using the solution casting method. A scanning electron microscope showed the voids and droplets, indicating the immiscibility of blends. Due to this immiscibility, the tensile strength values of the blends were found to be in between that of pure PBS (42.85 MPa) and pure PLLA (31.39 MPa). HAPs act as a compatibilizer by incorporating themselves in the voids and spaces caused by the immiscibility, thus increasing the overall tensile strength of the resulting composite to a certain extent, e.g., the tensile strength of PBS/PLLA = 95/5 loaded with 50 mg HAPs was found to be 51.16 MPa. The structural analysis employing the X-ray diffraction (XRD) patterns confirmed the formation of polymer blends and composites. The contact angle analysis showed that the addition of HAPs increased the hydrophilicity of the resulting composites. Selective samples were investigated based on mechanical properties to see if the blends and composites are biocompatible. The obtained results showed that all of the samples with better mechanical properties demonstrated good biocompatibility. This indicates the effectiveness of scaffolds for tissue regeneration.

## 1. Introduction

For the reconstruction and functional recovery of injured bone, researchers around the globe are working on bone tissue engineering by designing novel materials that are not only biocompatible, but also biodegradable [1,2,3,4,5]. However, despite the research in this field, non-biodegradable implants for regenerating injured bone are still in use. The incorporation of these permanent biomaterials for the reorganization of functional tissues causes them to stay in the body forever [6,7,8,9]. Nevertheless, this growing research field is in pursuit of finding a solution regarding biodegradable cum bioactive materials with mechanical and thermal properties comparable to those of bones. As far as the mechanical properties of bone tissues are concerned, the biomaterials should bear the same properties and should also favor the cellular functions, such as cell adhesion, proliferation, migration, and differentiation [10,11]. To achieve the required mechanical properties of polymers, different blends and composites are prepared, and surface modification is performed to achieve the desired surface and biological properties. Numerous hybrid composite biomaterials or scaffolds prepared from combinations of either natural or synthetic materials have been investigated to obtain the synergistic properties required for bone tissue engineering [3,12,13,14,15,16,17,18,19,20].

Biodegradable polymers have gained much importance in biomedical applications, especially aliphatic polyesters such as poly (l-lactic acid) PLLA and poly (butylene succinate) (PBS). PLA has been successfully used in bone fracture fixation and tissue engineering scaffolds and has exhibited various advantages over non-degradable materials, such as an absence of stress shielding effects, the lack of a need for re-surgery for the removal of implants, and its ability to be molded into any kind of shape [21,22,23,24,25]. PLLA material has been used for more than a decade in orthopedic surgery, owing to its good biocompatibility in applications such as bone substitutes, bone fixation, tendon reconstructions, and the repair of osteochondral defects and ligaments. Apart from being highly biocompatible, PLLA also displays favorable biophysical properties and is thus considered a good candidate for artificial subcutaneous prostheses and cardiovascular surgery applications [26]. On the other hand, PBS has shown promising potential applications in the biomedical industry due to its enhanced processability and biocompatibility, and its impact strength is similar to that of polyethylene and polypropylene. Moreover, the osteo-conductive properties of PBS have been verified by its better cellular adhesion and proliferation (both in vitro and in vivo) [27,28,29]. Most importantly, inside the body, PBS primarily degrades into succinic acid, with final harmless degradation products of H_2_O and CO_2_ [30]. However, PBS fails to meet all of the requirements due to its slow degradation rate and lower tensile strength [31].

Hydroxyapatite (HA) is a form of bio-ceramic material that is chemically similar to bones and hard tissues found in humans. HA is biocompatible with our biological system and does not cause any harm or toxicity. It is also the principal component of natural bone, with a chemical formula of Ca_10_(PO_4_)_6_(OH)_2_ and hexagonal structure. About 70% of bone is comprised of hydroxyapatite with a calcium to phosphorous ratio of 1.67. Due to its excellent biocompatibility, synthetic HA can be rapidly integrated into the human body [32,33]. Although this material is highly biocompatible, its application in the field of biomaterials is limited due to its fragility, low mechanical strength, easy rupture, and weak fatigue resistance [34]. For this reason, HA nanoparticles (HAPs) are used as a nano-filler in the polymer matrix for withstanding the load and to achieve synergistic properties of HA and the biopolymer. Eftekhari et al. [35] fabricated a PLLA/cellulose/HA nanocomposite that resulted in an improvement of the Young’s modulus value from 6.6 to 38 MPa (required for trabecular bone). Moreover, Huang et al. [36] investigated the degradation rate and mechanical strength of a PLLA/n-HA (nano-hydroxyapatite) composite compared to neat PLLA and found that the PLLA/n-HA material had enhanced properties compared to PLLA material for artificial bone. Furthermore, Li et al. [37] experimented with in vitro mineralization and cell culture of n-HA/PBS porous scaffolds, and from the results, found that these scaffolds have a good osteogenic capacity and cell compatibility. Similarly, a lot of research has been conducted on biodegradable polymer-based scaffolds; however, finding a suitable material that has all the properties of natural bone is still a major challenge.

In this study, we prepared PBS/PLLA/HAPs composites by the solution casting process. To the best of our knowledge, no work has previously been reported for this composite. By varying the composition of the HAP content in the PBS/PLLA blends, a range of HAP-reinforced PBS/PLLA composites were obtained and discussed. The mechanical properties and cell biocompatibility were also investigated and discussed.

## 2. Experimental Method

### 2.1. Chemicals and Materials

PLLA (MW–80,000–100,000; Polysciences Inc., Taipei, Taiwan), PBS (BioPBS, FZ91PM, PTT MCC Biochem Co. LTD., Bangkok, Thailand), calcium nitrate tetrahydrate (CA 0231; reagent grade, Scharlau, Barcelona, Spain), di-ammonium hydrogen phosphate (extra pure NF, Scharlau, Barcelona, Spain), ammonia solution (32%; Emplura Merck, Darmstadt, Germany), and chloroform (99-99.4: GC; Sigma Aldrich, Steinheim, Germany) were employed in this study.

### 2.2. Synthesis of Hydroxyapatite Nanoparticles (HAPs)

The stepwise synthesis of HAPs using the reported method [38] is as follows:

- A total of 130 g of calcium nitrate tetrahydrate (Ca(NO_3_)_2_·4H_2_O) was dissolved in 800 mL distilled water, followed by continuous stirring, while maintaining the temperature at 80 °C and pH at 12 by adding ammonia solution;
- Separately, 38 g of di-ammonium hydrogen phosphate ((NH_4_)_2_HPO_4_) was dissolved in 550 mL distilled water under similar conditions;
- Ca(NO_3_)_2_·4H_2_O solution was added drop wise to (NH_4_)_2_HPO_4_ solution and the obtained solution was stirred for 2 h at 80 °C;
- For the precipitation of HAPs, the obtained solution was left for 24 h at room temperature and afterwards, was repeatedly washed with distilled water;
- After washing, the solution was filtered and the obtained powder (HAPs) was dried at 80 °C for 24 h. The dried powder was calcined at 550 °C for 3 h.

**Figure 1:**
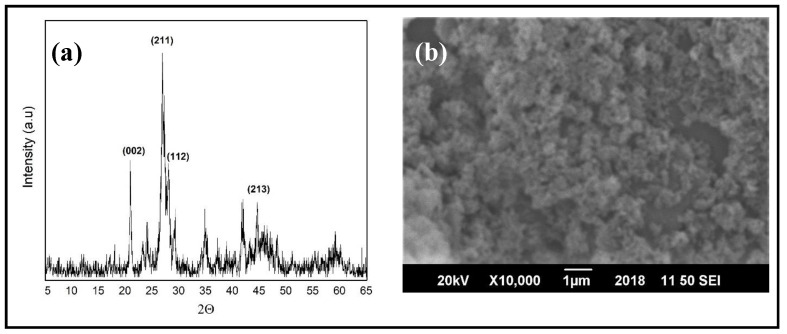
(**a**) X-ray diffraction (XRD) of synthesized hydroxyapatite nanoparticles (HAPs). (**b**) Scanning electron microscopy (SEM) image of the powder HAPs.

To confirm the structural formation of HAPs, X-ray diffraction (XRD) was performed, as shown in Figure 1a, and the diffraction peaks matched the reference pattern JCPDS Card No. 09-0432 for HA. For the morphological studies, scanning electron microscopy (SEM) was performed, as shown in Figure 1b. The morphology obtained depicted the agglomeration of spherical-shaped HAPs. The size of the nanoparticles was found to be around 80 ± 20 nm.

### 2.3. Preparation of PBS/PLLA Polymer Blends

To prepare the polymer blends of polybutylene succinate (PBS) and poly (l-lactic acid) (PLLA), the solution casting method was employed. Both polymers were taken in different amounts (total amount taken as 1 g) to form various concentrations with respect to each other, i.e., PBS/PLLA = 100/0, 95/5, 90/10, 85/15, 80/20, 70/30, 50/50, and 0/100 in weight fraction. The respective amounts of each polymer were dissolved in 15 mL of chloroform separately. The solutions were stirred continuously for 24 h so that the polymers were completely dissolved. Both solutions were mixed and stirred again for 24 h so that a uniform blend of both polymers was obtained. The blend was cast in a Petri dish and dried at 50 °C for 4 h in an oven. Upon the completion of drying, a uniform film of polymer blend with a 0.1 mm thickness was obtained.

### 2.4. Preparation of the PBS/PLLA/HAP Composite

To prepare the composite of a previously obtained polymer blend with hydroxyapatite nanoparticles (HAPs), an extra step was introduced before the drying of the blend. Different amounts of HAPs, i.e., 10, 20, 50, and 100 mg, were dispersed in 10 mL chloroform along with 10 mg of SDS (coupling agent). This dispersion was also stirred for 24 h and then added to the polymer blend solutions. The solution obtained was further stirred for 24 h, followed by the same steps of drying and casting mentioned above. The composite film obtained was, for example, 95/5/10, i.e., in PBS/PLLA (95/5) blend, 10 mg of HAPs was added. The same nomenclature was followed while designating all of the obtained composite films.

### 2.5. Characterization

Investigations of the surface properties and biological response were performed on the upper surface of the obtained films. The samples obtained from these films were characterized by the following techniques.

The tensile properties of polymer blends and nanocomposites were tested according to ASTM D882 on a universal testing machine (Shimadzu AGX Plus, Kyoto, Japan) at a constant crosshead speed of 1 mm/min and using a load cell of 20 kN. The dimensions of the tensile specimen were 2 cm × 1 cm × 0.1 mm (L × W × T). The tensile strength data were obtained by averaging five specimens of each sample and the results are reported as the mean ± SD.

The surface morphology of polymer blends and the PBS-PLLA-HAP composite was investigated by SEM studies carried out on the Jeol JSM-6490A (Tokyo, Japan).

XRD analysis was performed by using a Siemens D5005 STOE & Cie GmbH (Darmstadt, Germany) manufactured XRD machine using a Cu Kα (λ = 0.15418 nm) radiation source operating at a current of 40 mA and voltage of 40 KV.

The static contact angle of the as-prepared samples was measured by a Kruss DSA 25 (Hamburg, Germany) Goniometer using a sessile drop (water) at room temperature (20 °C) for 0–20 s. The contact angle data were obtained by averaging five specimens of each sample and the results are reported as the mean ± SD.

The Hela cell line was obtained from ATCC (Manassas, VA, USA) and mCherry-ER was obtained from addgene (Watertown, MA, USA). Hela cells were used as a model system to check the biocompatibility and general response of the polymer, blends, and composite samples against the cell line. mCherry labeled Endoplasmic Reticulum (ER) was cultured and maintained in DMEM media at 37 °C under normal humidity. The samples were sterilized using ethanol, washed by Phosphate Buffer Saline (PBS), and finally dried before cell seeding. Sterilized selected polymer blends and composites were placed in 24-well plates and cells in suspension were added on the top. The cells were grown on the samples for 24 h and transferred to a fresh plate, fixed with 4% PFA for 8 min at room temperature. The samples were washed with PBS twice and then subjected to imaging. The images were obtained using Axio Imager A1 (Zeiss microscope, Oberkochen, Germany), with a 20X objective. For cell viability measurement, the cells were incubated at room temperature for 30 min and transferred to a fresh plate, and 400 μL of media from the old plate was added. Cells were incubated with 200 µL of CellTiter-Glo (R) (Promega G7570, Wis, USA) and shaken for 10 min, and the luminescence was then measured using a plate reader. The mean cell viability was calculated from three independent experiments and statistical analysis was performed using the ANOVA model.

## 3. Results and Discussion

**Figure 2:**
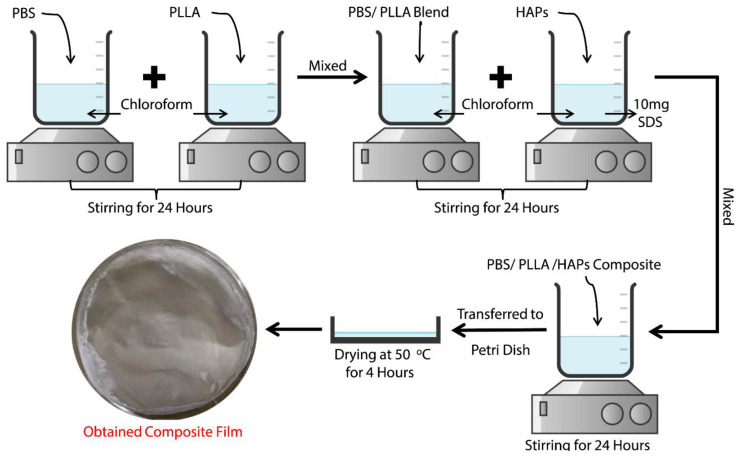
Scheme of the solution casting method for HAP-reinforced poly (butylene succinate) (PBS)/poly (l-lactic acid) (PLLA) polymer blend composites (PBS-PLLA-HAPs).

Our study demonstrates a solution casting procedure for the preparation of composite scaffolds of PBS/PLLA polymer blends with hydroxyapatite nanoparticles for orthopedic application. Figure 2 presents a schematic procedure of the PBS/PLLA/HAP composite.

### 3.1. Mechanical Properties of PBS/PLLA Polymer Blends and PBS/PLLA/HAP Composites

**Figure 3:**
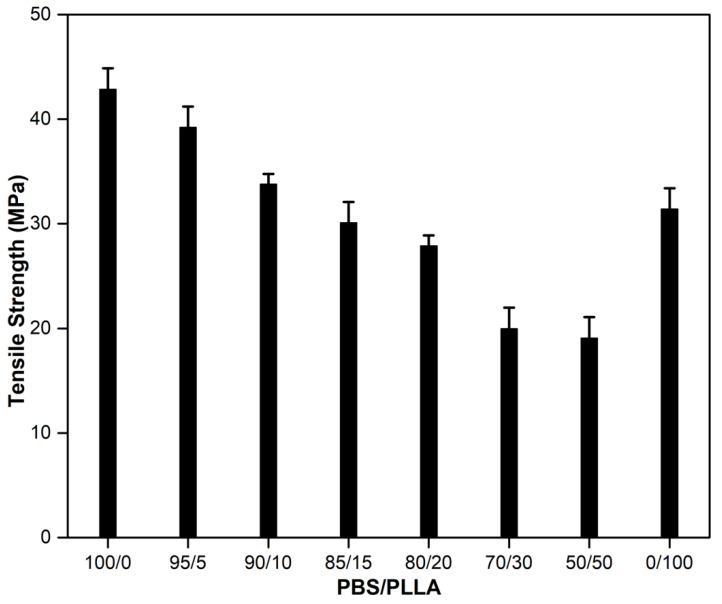
Tensile strength values of PBS-PLLA polymer blends.

The tensile strength of the PBS-PLLA polymer blends is given in Figure 3. The tensile strength of pure PBS (42.85 MPa) is higher than that of pure PLLA (31.39 MPa). The addition of different wt% of PLLA to pure PBS reduces the tensile strength because the two phases are immiscible with each other [39]. The greater the addition of PLLA, the more the immiscibility results in the generation of stress concentrators at the interface of the two polymers, resulting in crack initiation. In this situation, the mechanical properties of the blends are decreased. As it is evident from Figure 3 that the tensile strength value for PBS/PLLA = 80/20, 70/30, and 50/50 has not improved much, no further treatments and characterizations were performed for them. Since polymers are immiscible, there is likely no interface/bonding between the two phases. HAPs are incorporated in these spaces and act as a compatibilizer, thus increasing the overall tensile strength of the blend to a certain extent. After reaching their threshold limit, HAPs start to make a separate phase and since hydroxyapatite is brittle in nature [40], its presence as a separate phase will result in the generation of a prominent ‘stress concentration area’, causing an overall decrease in the tensile strength. To evaluate the effect of HAPs on the overall tensile strength of the PBS-PLLA polymer blends, HAPs in different amounts (10, 20, 50, and 100 mg) were loaded in PBS/PLLA = 95/5, 90/10, and 85/15 to prepare the PBS-PLLA-HAP composites. The tensile strength of the PBS-PLLA-HAP composites is given in Table 1.

As is evident from Table 1, with the 85/15 blend, HAP loadings did not result in an improvement of the tensile properties, while 95/5 and 90/10 blends showed a considerable increase in the overall tensile strength of the composite. The reason behind this result may be that, since PBS and PLLA are immiscible, as long as both polymers are equal in ratio, immiscibility will result in the generation of larger spaces for the HAPs to fill at the interface of the two polymers. If the amount of HAPs increases in a certain area, they will agglomerate, causing a separate brittle phase to grow in the composite. This brittle phase decreases the tensile properties of the overall composite. From the overall results of the mechanical properties, we concluded that keeping the PBS/PLLA weight ratio at around 90-99/10-1 and 50 mg HAP loading can greatly enhance the overall mechanical properties of the PBS-PLLA-HAP composite to be used as scaffold for bone tissue engineering.

### 3.2. Morphological Studies of PBS/PLLA Polymer Blends and PBS/PLLA/HAP Composites

**Figure 4:**
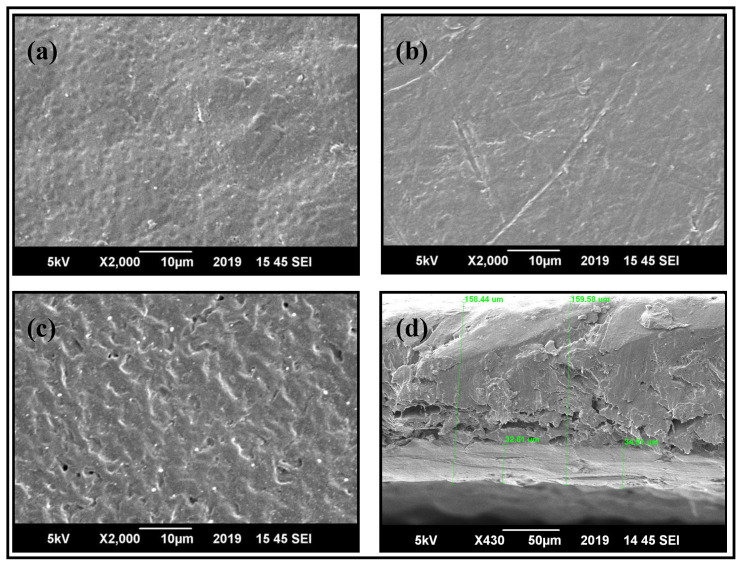
SEM images of (**a**) pure PLLA, (**b**) pure PBS (**c**), the 95/5 blend, and (**d**) the cross section of the 95/5 blend.

SEM was used to analyze the surface morphology of base polymers, polymer blend samples, and HAP-incorporated polymer blends. Figure 4a,b shows the surface morphology of pure PLLA and pure PBS, respectively, while Figure 4c,d depicts the surface of PBS/PLLA = 95/5 and its cross section, respectively. Pure polymers were flat and had mostly smooth surfaces with no signs of pores; however, in the 95/5 blend, voids and droplets were quite visible on the surface, as well as in the cross section. These voids indicate the immiscibility of the blend. The same morphological behavior was observed in the rest of the blends.

**Figure 5:**
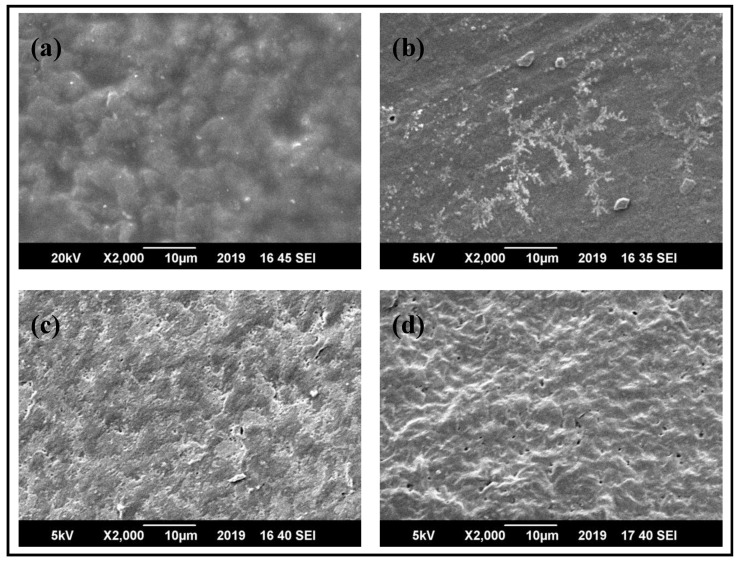
SEM images of PBS/PLLA = 95/5 with (**a**) 10 mg HAPs, (**b**) 20 mg HAPs, (**c**) 50 mg HAPs, and (**d**) 100 mg HAPs.

In the HAP-incorporated polymer blend composites, e.g., PBS/PLLA = 95/5, as shown in Figure 5, SEM micrographs revealed that the HAPs were randomly distributed in the polymer blends. At increased concentrations of HAPs in the polymer blends, the presence of HAP filler can be indicated by bright white spots in the micrographs. It was observed that at lower concentrations of HAPs, such as 10 and 20 mg in the matrix, their dispersion is quite homogenous, with the least agglomeration. At lower concentrations of HAPs, no visible cracks were found on the surfaces of the polymer blend matrix, indicating good adhesion between the polymer blend matrix and HAP filler material. At higher concentrations of HAPs, voids start forming inside the matrix material. This is because, when there is a higher concentration of HAPs in the matrix, HA to HA interaction increases (causing agglomeration), thus decreasing the interaction of filler with the matrix. This leads to the detachment of HAPs from the matrix, causing a decline in the mechanical properties at higher concentrations of HAPs in the matrix. Almost the same trend was observed in HAPs incorporated in PBS/PLLA = 90/10 and 85/15.

### 3.3. Structural Studies of PBS/PLLA Polymer Blends and PBS/PLLA/HAP Composites

**Figure 6:**
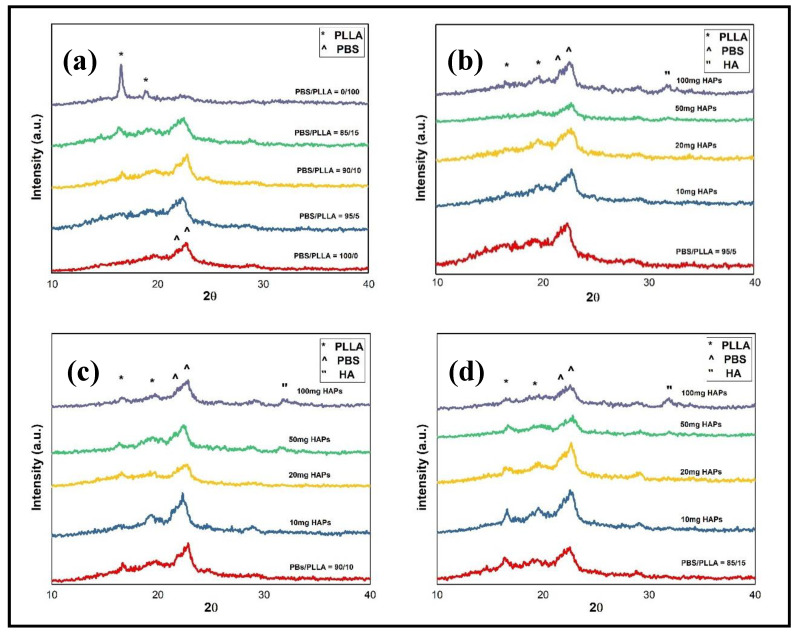
XRD of (**a**) polymer blends, (**b**) HAPs incorporated in PBS/PLLA = 95/5, (**c**) HAPs incorporated in PBS/PLLA = 90/10, and (**d**) HAPs incorporated in PBS/PLLA = 85/15.

To determine the structural formation of polymer blends and composites, XRD analysis was performed. In Figure 6a, XRD plots of pure PBS; pure PLLA; and PBS/PLLA = 95/5, 90/10, and 85/15 polymer blends are shown. The peaks of PBS and PLLA identified are marked accordingly. The PLLA peak at 2θ = 17 and 19 corresponds to (110) and (203) planes, respectively. Similarly, the PBS peak at 2θ = 21.5 and 22.4 corresponds to (021) and (110) planes, respectively. The XRD results for polymer blends show that the peaks of both the polymers are evident and as the PLLA composition increases, PLLA peaks become more prominent and their intensity also increases. In Figure 6b–d, the HAP peak at 2θ = 32 corresponds to the (211) plane and it is clear that as HAP loading increases to 100 mg, the HAP peak becomes more pronounced, confirming the formation of a separate phase of HAPs in the composite. Up to 50 mg loading, HAPs are mostly dispersed homogeneously in the polymer blends. The XRD patterns confirm the formation of PBS/PLLA/HAP composites.

### 3.4. Contact Angle Analysis of PBS/PLLA Polymer Blends and PBS/PLLA/HAP Composites

**Figure 7:**
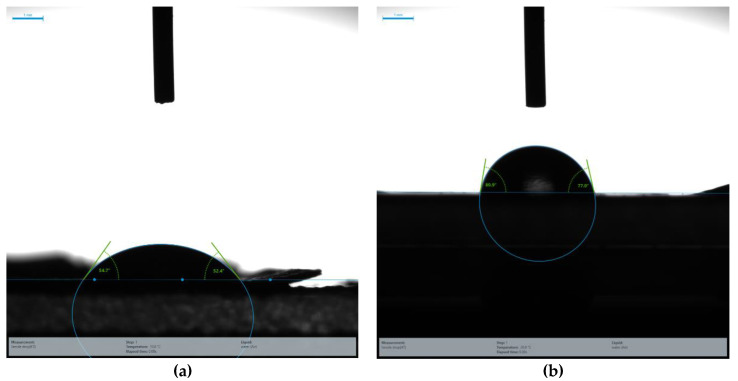
Contact angle: (**a**) Pure PLLA and (**b**) pure PBS.

**Figure 8:**
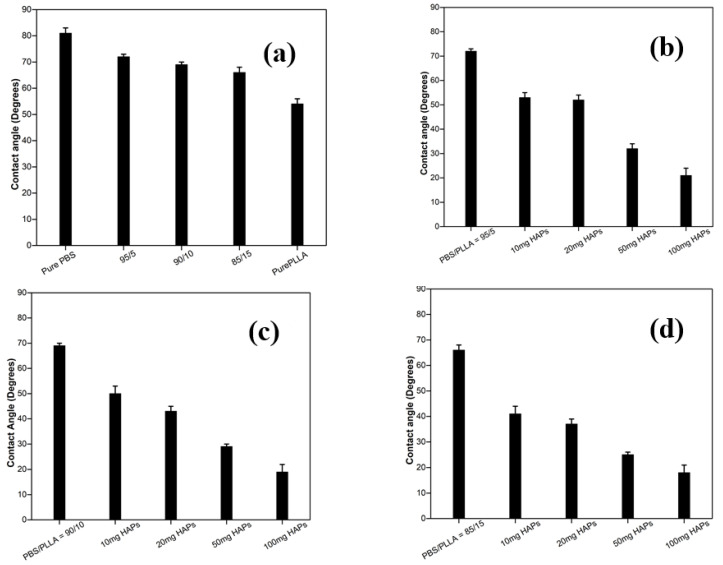
Values of the contact angle: (**a**) Polymer blends, (**b**) HAPs incorporated in PBS/PLLA = 95/5, (**c**) HAPs incorporated in PBS/PLLA = 90/10, and (**d**) HAPs incorporated in PBS/PLLA = 85/15.

The surface wettability of scaffolds used for bone tissue engineering should be intensively studied as, for cell growth and proliferation, the surface should be hydrophilic [41]. The surface wettability of polymer blends and composites was studied by measuring the static contact angle with a sessile drop of distilled water deposited on the sample surface. Surface wetting was examined by measuring the contact angle formed between the water drop and the surface of samples. It was observed that the PLLA (54°) is more hydrophilic than the PBS (80°), as Figure 7 and Figure 8a show that, as the composition of PLLA is increased in PBS, the contact angle values exhibit a gradual decrease. HA is hydrophilic and with the addition of different amounts of HAPs in the polymer blend, the contact angle value further decreased. As this criterion is followed, pure PBS should have the highest contact angle value, while 100 mg HAP-loaded PBS/PLLA = 85/15 should have lowest contact angle value. The results shown in Figure 8a–d satisfy this criterion very well; the value of the contact angle for pure PBS is 80°, while the value of the contact angle for 100 mg HAP-loaded PBS/PLLA = 85/15 is 18°. The presence of HAPs in the polymer blend leads to increasing water absorption and a hydrophilic character of the PBS/PLLA/HAP composite, which would favor good cell growth, proliferation, and viability in these materials [42].

### 3.5. Biological Response of PBS/PLLA Polymer Blends and PBS/PLLA/HAP Composites

**Figure 9:**
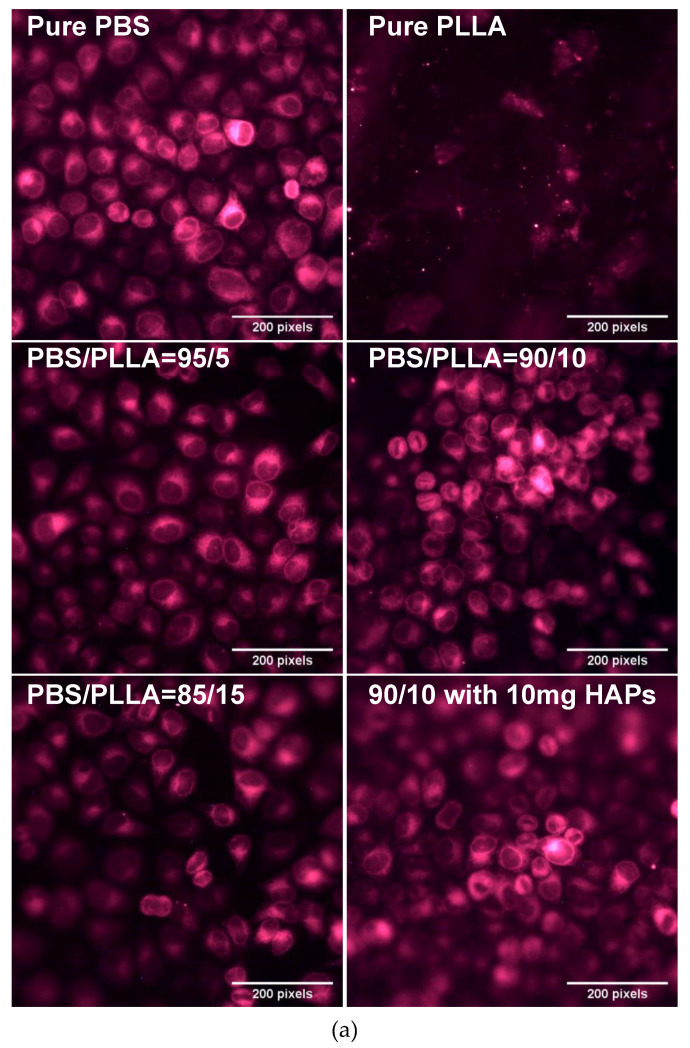

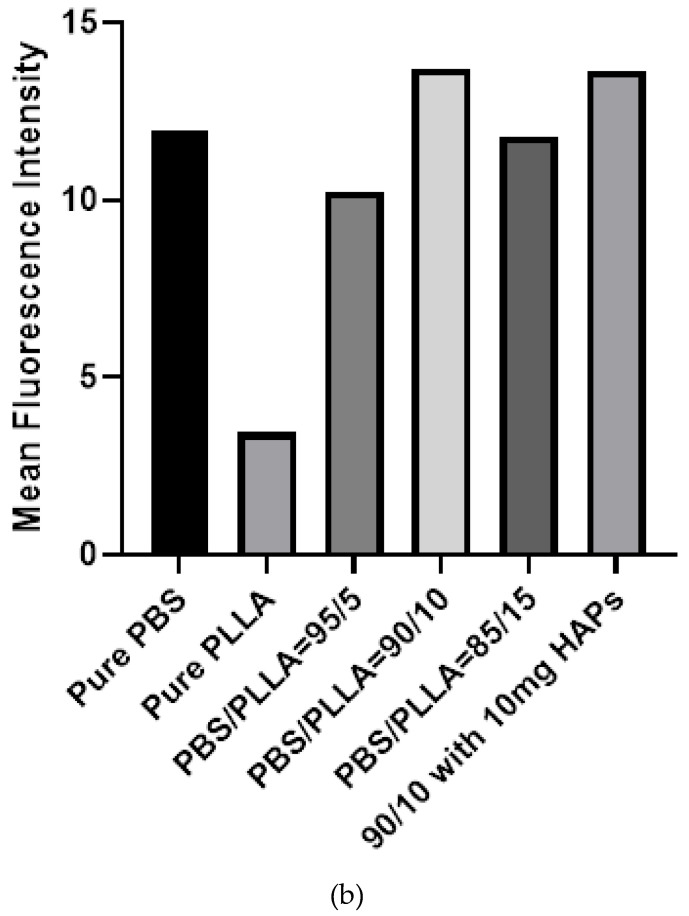
(**a**) Endoplasmic reticulum stained cells cultured on different samples, including pure PBS, PLLA, blends, and composites. (**b**) Quantification of the fluorescence intensity of endoplasmic reticulum stained cells cultured on different samples, including pure PBS.

The endoplasmic reticulum (ER) is the most dynamic and abundant organelle of cells, spreading throughout them. ER responds to any stress at the earliest point in time and thus serves as an efficient marker for cellular stress culture in biomaterial with different stiffnesses. Selective samples were investigated based on the mechanical properties to see if the blends and composites are biocompatible. Figure 9a shows less viable cells on pure PLLA, whereas the PBS surface demonstrated healthy viable cells. Following this, the cells were cultured on PBS/PLLA = 95/5, 90/10, and 85/15 polymer blends and all of these materials displayed good biocompatibility with normal cell shapes. Finally, the composite with the best mechanical properties, i.e., PBS/PLLA = 90/10 with 10 mg HAP loading, was also investigated and exhibited healthy cells on the surface. As stated earlier, a surface that exhibits more cellular stress negatively responds to cells. Therefore, a composite surface with healthy and viable cells demonstrates that the cells are not experiencing any stress. Moreover, the presence of HAPs would show osteo-conduction and help in cell differentiation, as per reported literature. As far as tissue regeneration is concerned, biocompatibility and cell viability on the surface of a scaffold represent the first step. Here, the results show that all of the samples with better mechanical properties demonstrated good cell viability. This not only indicates the biocompatibility of the scaffolds, but also its effectiveness for tissue regeneration. The fluorescence was quantified using image J software and plotted in Figure 9b to present the quantified data. Figure 9b also demonstrates that the selective samples that were tested were all biocompatible and hence can be further characterized for their detailed biological response.

**Figure 10:**
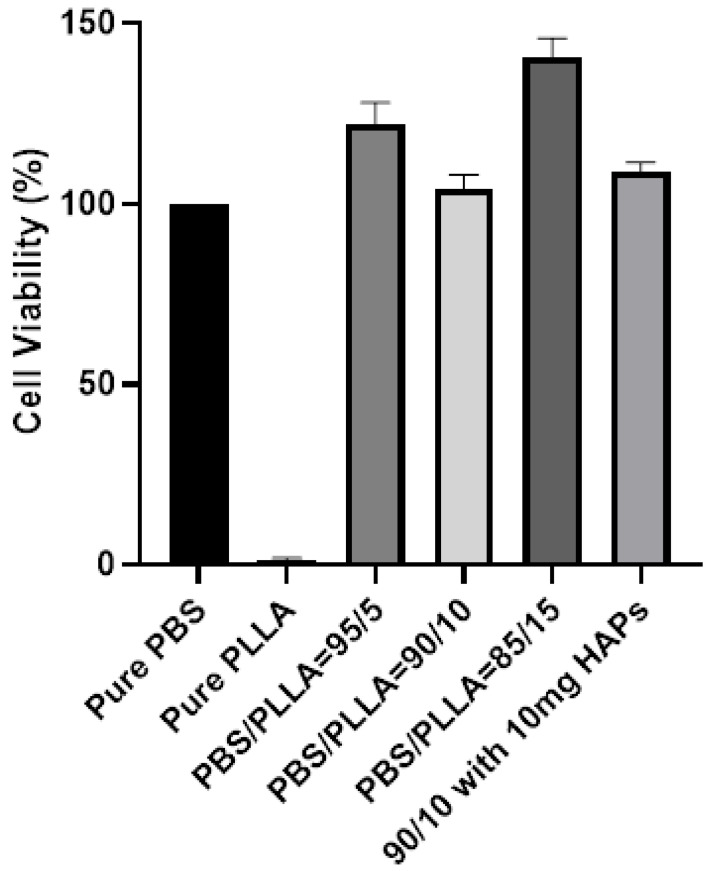
Cell viability results of pure polymers, polymer blends, and PBS/PLLA = 90/10 with 10 mg HAP loading. The graph represents three independent replicates. Only the PLLA to PBS difference is significant (*p* value = 0.001) and the rest are non-significant compared to PBS.

The data of CellTiter-Glo(R) for all of these samples are also presented in Figure 10. The quantitative data demonstrate and validate the results presented in Figure 9a,b. Figure 9 (both (a) and (b)) shows that all of the scaffolds that were tested were biocompatible and cells were viable in terms of growth. Pure PLLA showed less cell viability in the first 24 h and the same can be seen in Figure 9a,b.

## 4. Conclusions

Scaffolds of PBS/PLLA/HAP composites were prepared by the solution casting method for an investigation of the improved mechanical properties and biocompatibility. SEM indicated that the PBS/PLLA blends are immiscible, resulting in the formation of voids. The loading of different amounts of HAPs in these blends filled the voids and thus improved the overall mechanical properties of the resulting composites to a certain threshold limit. From the overall results of the mechanical properties, it was concluded that keeping PBS/PLLA wt% at around 90-99/10-1 and 50 mg HAP loading can greatly enhance the overall mechanical properties of the PBS/PLLA/HAP composite to be used as a scaffold for bone tissue engineering. Hela cells, when cultured on the selective samples, demonstrated excellent biocompatibility and cell attachment on the polymer blends loaded with HAPs. This study was only focused on determining the biocompatibility and cell attachment of the scaffolds. Following this study, cell proliferation and differentiation can be studied in the future to better understand the relationship between the scaffolds and their capability for tissue regeneration.

## Figures and Tables

**Table 1:** Tensile strength values of PBS-PLLA-HAP composites.

PBS/PLLA	HAPs (mg)	Tensile Strength (MPa) (Mean ± SD)
**95/5**	0	39.2 ± 2.0
	10	39.9 ± 2.8
	20	39.0 ± 1.7
	50	51.2 ± 1.3
	100	43.1 ± 0.8
**90/10**	0	33.8 ± 1.0
	10	56.9 ± 1.4
	20	45.0 ± 2.0
	50	23.9 ± 2.8
	100	29.6 ± 1.5
**85/15**	0	30.1 ± 2.1
	10	26.2 ± 1.9
	20	27.9 ± 0.2
	50	27.5 ± 1.1
	100	17.8 ± 2.9
